# The Antifungal Itraconazole Is a Potent Inhibitor of Chikungunya Virus Replication

**DOI:** 10.3390/v14071351

**Published:** 2022-06-21

**Authors:** Lucca R. Policastro, Isabela Dolci, Andre S. Godoy, José V. J. Silva Júnior, Uriel E. A. Ruiz, Igor A. Santos, Ana C. G. Jardim, Kirandeep Samby, Jeremy N. Burrows, Timothy N. C. Wells, Laura H. V. G. Gil, Glaucius Oliva, Rafaela S. Fernandes

**Affiliations:** 1 São Carlos Institute of Physics, University of São Paulo, São Carlos 13563-120, Brazil; lrpolicastro@gmail.com (L.R.P.); isabela@estudante.ufscar.br (I.D.); andregodoy@ifsc.usp.br (A.S.G.); 2 Virology Sector, Department of Preventive Veterinary Medicine, Federal University of Santa Maria, Santa Maria 97105-900, Brazil; valter_science@hotmail.com; 3 Virology Sector, Laboratory of Immunopathology Keizo Asami, Federal University of Pernambuco, Recife 50670-901, Brazil; 4 Institute of Biomedical Sciences, Federal University of Uberlândia, Uberlândia 38405-302, Brazil; uriel.aquino79@gmail.com (U.E.A.R.); igoras244@gmail.com (I.A.S.); jardim@ufu.br (A.C.G.J.); 5 Institute of Biosciences, Humanities and Exact Sciences (Ibilce), São Paulo State University (UNESP), Campus São José do Rio Preto, São Josédo Rio Preto 15054-000, Brazil; 6 Medicines for Malaria Venture, P.O. Box 1826, 1215 Geneva, Switzerland; sambyk-consultants@mmv.org (K.S.); burrowsj@mmv.org (J.N.B.); wellst@mmv.org (T.N.C.W.); 7 Aggeu Magalhães Institute, IAM-FIOCRUZ, Recife 50670-420, Brazil; lgilfiocruz@gmail.com

**Keywords:** Chikungunya virus, replicon-based assays, drug development, antiviral, Itraconazole

## Abstract

Chikungunya virus (CHIKV) is the causative agent of chikungunya fever, a disabling disease that can cause long-term severe arthritis. Since the last large CHIKV outbreak in 2015, the reemergence of the virus represents a serious public health concern. The morbidity associated with viral infection emphasizes the need for the development of specific anti-CHIKV drugs. Herein, we describe the development and characterization of a CHIKV reporter replicon cell line and its use in replicon-based screenings. We tested 960 compounds from MMV/DNDi Open Box libraries and identified four candidates with interesting antiviral activities, which were confirmed in viral infection assays employing CHIKV-*nanoluc* and BHK-21 cells. The most noteworthy compound identified was itraconazole (ITZ), an orally available, safe, and cheap antifungal, that showed high selectivity indexes of >312 and >294 in both replicon-based and viral infection assays, respectively. The antiviral activity of this molecule has been described against positive-sense single stranded RNA viruses (+ssRNA) and was related to cholesterol metabolism that could affect the formation of the replication organelles. Although its precise mechanism of action against CHIKV still needs to be elucidated, our results demonstrate that ITZ is a potent inhibitor of the viral replication that could be repurposed as a broad-spectrum antiviral.

## 1. Introduction

Chikungunya virus (CHIKV) is an alphavirus first appeared in 1952, which causes periodic and explosive outbreaks of chikungunya fever [1]. The symptoms of this disease include fever, headache, myalgia, and severe polyarthritis that could persist for years [2,3]. In February 2005, a major CHIKV outbreak occurred on the Indian Ocean islands, followed by a high number of infection cases reported in Europe and India in 2006 and 2007, respectively [4,5]. In December 2013, CHIKV emerged in Caribbean, and by the end of December 2015 about 1 million cases had been notified in the Americas [6,7]. Although the most recent outbreak was reported in February 2018 in Mombasa, Kenya, the reemergence of the virus in many parts of the world represents a serious public health concern [8]. Up to date, no CHIKV-specific antiviral therapy has been approved, being the treatment palliative, to alleviate the symptoms [9].

CHIKV is an enveloped virus with a positive-sense single stranded RNA genome of approximately 12 kb in length, containing two open reading frames (ORFs) flanked by 5′ and 3′ untranslated regions (UTRs) and separated by a non-coding intergenic region. The first ORF encodes a non-structural polyprotein produced by direct translation of the genome, which is proteolytically processed into four non-structural proteins (nsP1-nsP4) [10]. The second ORF encodes five structural proteins (C, E3, E2, 6K, and E1), which are expressed from a subgenomic RNA [11]. The nsPs assemble to form the viral replication complex, which is responsible for RNA synthesis, while the structural proteins assemble into new viral particles [2].

Viral replicon systems, which express all the genetic elements required for their own replication without producing infectious particles, have been widely used as an alternative tool in the search for antiviral drugs [12,13]. In the last decade, CHIKV replicon cell lines have been developed and used to screen inhibitors of the viral replication [14,15,16,17,18,19]. Among the hit compounds identified, abamectin, ivermectin, and berberine, showed significant inhibitory activities [16]. Berberine, an isoquinoline alkaloid, was shown to reduce virus-induced activation of all major MAP kinase pathways in a follow-up study, and also demonstrated to be effective in alleviating CHIKV-induced inflammatory symptoms in a mouse model [20].

In this study, we describe the development of a stable CHIKV replicon cell line, the BHK-21-T7-Gluc-nSP-CHIKV-99650, which harbors a replicative replicon expressing *Gaussia* luciferase (GLuc) as a reporter gene. Using this cell line in replicon-based assays, we evaluated three MMV/DNDi small-molecule libraries, the Pandemic Response (PRB), Pathogen, and COVID boxes, all containing molecules either marketed or in development, with known or predicted antiviral, antifungal, or antibacterial activities [21,22,23], for the identification of anti-CHIKV agents. From the tested molecules, the antifungal itraconazole was the most effective, exhibiting a selectivity index (SI) of > 312. Additionally, its antiviral activity was confirmed in viral infection assays (SI > 294), showing that this molecule is a potent inhibitor of CHIKV replication in vitro.

## 2. Methods

### 2.1. Cells, Virus, and Compounds

BHK-21 cells, from the Global Bioresource Center (ATCC),were maintained in Dulbecco’s Modified Eagle’s Medium (DMEM, Sigma-Aldrich, St. Louis, MI, USA) supplemented with 100 U/mL of penicillin 100 mg/mL of streptomycin, 1% dilution of stock of non-essential amino acids (HYCLONE Laboratories, Logan, UT, USA) and 10% of fetal bovine serum (FBS, HYCLONE Laboratories, Logan, UT, USA) in a humidified 5% CO_2_ incubator at 37 °C. BHK-21-GLuc-nSP-CHIKV-99650 cells, from the Laboratório de Virologia e Terapia Experimental (LaViTE-Aggeu Magalhães Institute, Recife, Brazil), were maintained in DMEM 10% FBS with 500 µg/mL geneticin (G418-Sigma-Aldrich, St. Louis, MI, USA).The CHIKV expressing *nanoluciferase* reporter (CHIKV-*nanoluc*) [24] used for the viral infection assays was based on the CHIKV isolate LR2006OPY1 (East/Central/South African genotype) and was produced, rescued, and titrated as previously described [25,26]. MMV/DNDi compounds (>90% purity) solubilized in 100% DMSO (*v*/*v*) were further diluted with assay media to a final DMSO concentration of up to 1% (*v*/*v*) for the assays. Suramin (Sigma-Aldrich, St. Louis, MI, USA) was solubilized in 100% DMSO at 20 mM and further diluted in assay media to a final concentration of 10 µM 1% DMSO (*v*/*v*). Itraconazole was purchased as a racemic mixture (CAS number: CAS-84625-61-6).

### 2.2. Construction of Rep-GLuc-nsP-CHIKV-99659

We constructed a CHIKV reporter subgenomic replicon, termed rep-GLuc-nsP-CHIKV-99659, by recombining four partially homologous fragments: **(i)** fragment 1, covering the T7 RNA polymerase promoter inserted by PCR amplification, and the 5′ UTR and nsP1-nsP4 amplified from the chemically synthesized CHIKV 99659 genome (GenScript, Piscataway, NJ, USA) (GenBank KJ451624); **(ii)** fragment 2, containing the CHIKV subgenomic promoter inserted by PCR amplification, and GLuc amplified from pGLuc-NS (WF10) (kindly provided by Dr. Daniel Perez, University of Georgia, Athens, GA, USA); **(iii)** fragment 3, the ubiquitination sequence and neomycin phosphotransferase (Neo) gene amplified from pBSC-YFV17D-LucNeoIres; and **(iv)** fragment 4 (3′UTR), amplified from the CHIKV 99659 genome (GenScript, Piscataway, NJ, USA).

The four fragments were amplified with Phusion^®^ High-Fidelity DNA Polymerase (New England Biolabs), using the oligonucleotides shown in Table 1 and recombined, in a single-cloning-step, into the pBSC-HDR shuttle vector (Gil et al., unpublished data) previously digested (*Bam*HI nuclease, New England Biolabs) and dephosphorylated (Alkaline Phosphatase, Calf Intestinal, CIAP, Promega, Madison, WI, USA).

Homologous recombination was performed in *Saccharomyces cerevisiae* (strain RFY 206, *Mata his3*Δ*200 leu2-3 lys2*Δ*201 ura3-52 trp1*Δ*::hisG*) [27] transformed by lithium acetate (LiAc) [28]. Colonies were screened in Yeast Nitrogen Base (YNB) without tryptophan [29] and cloning was confirmed by PCR, using the oligonucleotides CHIKV-3′UTR-F and pBSC-SpeI-3′CHIKV-R (Table 1). Finally, *Escherichia coli* (strain DH10B) (Invitrogen, Waltham, MA, USA) was transformed with the positive clones [29] and plasmid DNA was extracted (QIAGEN Plasmid Midi Kit, QIAGEN, Germantown, MD, USA) and used as described below.

### 2.3. Full-Length PCR and In Vitro Transcription

The rep-GLuc-nsP-CHIKV-99659 sequence was linearized and amplified from the DNA plasmid by full-length PCR, using AccuTaq^™^ LA DNA Polymerase (Sigma) and the oligonucleotides pBSC-BamHI-T7Phi2.5-5′CHIKV-F and CHIKV-3′UTR-R (Table 1). Amplicons were purified by UltraPure™ Phenol: Chloroform: Isoamyl Alcohol (Invitrogen), precipitated with ethanol and sodium acetate (3M), and used as template for in vitro transcription using the T7 RiboMAX™ Express Large Scale RNA Production System-T7 (Promega, Madison, WI, USA).

### 2.4. Cell Transfection and Development of the BHK-21-GLuc-nsP-CHIKV-99659 Cell Line

BHK-21 cells were transfected with the in vitro transcribed RNA. Briefly, 2 × 10^6^ cells were resuspended in 100 μL of cytomix buffer (120 mM KCl, 0.15 mM CaCl_2_, 10 mM K_2_HPO_4_/KH_2_PO_4_ pH 7.6, 25 mM HEPES pH 7.6, 2 mM EGTA, 5 mM MgCl_2_) with 2 mM ATP and 5 mM glutathione, and electroporation was performed in 2 mm cuvette with 140 V and 25 msec pulse (Gene Pulser Xcell, Bio-Rad, Hercules, CA, USA). Three days post-transfection cells were selected in medium supplemented with 700 μg/mL Geneticin^®^ (Gibco, Waltham, MA, USA), and after ten days, cell colonies were removed by Scienceware^®^ cloning discs (Sigma-Aldrich, St. Louis, MI, USA) soaked in trypsin (Gibco, Waltham, MA, USA), seeded individually and amplified in medium with Geneticin^®^ (500 μg/mL). The selected cell line was denominated BHK-21-GLuc-nsP-CHIKV-99659.

### 2.5. Stability Analysis of BHK-21-GLuc-nsP-CHIKV-99659

Parental and BHK-21-GLuc-nsP-CHIKV-99659 cells (at Passage 3 and 13) were seeded in duplicates in a 96-well plate (10^5^ cells/well). Eighteen hours after seeding, 10 μL of the supernatant from each culture was collected and GLuc activity measured using the BioLux Gaussia Luciferase Assay Kit (New England Biolabs, Ipswich, MA, USA) in Mithras LB 940 Multimode Microplate Reader (Berthold Technologies, Bad Wildbad, Germany). Relative light unit (RLU) values of BHK-21-GLuc-nsP-CHIKV-99659 (at p3 and p13) were represented in fold-increase compared to the negative control (parental BHK-21 cell).

### 2.6. Validation of Replicon-Based Assays Using Suramin

The validation of the replicon-based assays was performed with the anti-parasitic drug suramin, a known inhibitor of CHIKV [19], as previously detailed in [30], with the exception that the GLuc signals were measured from the supernatant of cells (40 µL were mixed with 100 μL of *Renilla* luciferase Assay Reagent [Promega, Madison, WI, USA]). To determine the cytotoxicity of suramin, we performed a MTT (3-(4,5-dimethylthiazol- 2-yl)-2,5-diphenyltetrazolium bromide) assay, as described in [30]. The compound concentration required to inhibit 50% of the GLuc activity (EC_50_) and cause 50% cytotoxicity (CC_50_) was estimated using the OriginPro 9.0 software. Two independent assays were performed in duplicates.

### 2.7. Replicon-Based High-Throughput Screening of MMV/DNDi Libraries

The COVID Box, PRB, and Pathogen Box were screened using the BHK-21-GLuc-nSP-CHIKV-99659 cells. Each compound was tested at 10 µM 1% DMSO for primary screenings in a 96-well HTS format. 1% DMSO (0% inhibition) and suramin (100% inhibition) were used as negative and positive controls, respectively. Statistical analysis of the data were made through the determination of Z’-values, as described in [30]. In parallel, the toxicity of the compounds were evaluated at the same concentration to exclude false-positive hits [12,30]. Compounds that showed inhibition of luciferase activity in ≥80% and were not toxic to the cells (≥80% cell viability) were evaluated in a concentration-dependent manner to determine their EC_50_ and CC_50_ values, both used to calculate the selectivity index (SI = CC_50_/EC_50_). The concentration–response curves were performed twice in duplicates for the selected compounds at a 2-fold or 5-fold serial dilutions, and the CC_50_ and EC_50_ values were calculated as described above (Item 2.6).

### 2.8. Viral Infection Assays with CHIKV-Nanoluc

To further characterize the antiviral activity of each compound, BHK-21 cells were seeded at a density of 5 × 10^4^ cells/well into 48-well plates for 24 h and infected with CHIKV-*nanoluc* (MOI of 0.1 PFU/cell) in the presence of ITZ, GSK-983, rubitecan, and MMV676270, serially diluted (from 10 to 0.078 µM for ITZ or 100 to 0.78 µM for the other three compounds). At 16 h post-infection (h.p.i.) samples were harvested, virus replication levels were quantified by luminescence using *Renilla* luciferase Assay Reagent [Promega, Madison, WI, USA], and cell viability was measured, as described in [25,26]. The effective and cytotoxic concentrations (EC_50_ and CC_50_, respectively) were calculated using OriginPro 9.0 software and used to determine the selectivity index [25,26]. Assays were performed twice in triplicates.

## 3. Results

### 3.1. Development, Characterization, and Validation of a CHIKV GLuc Replicon Cell Line

**Figure 1:**
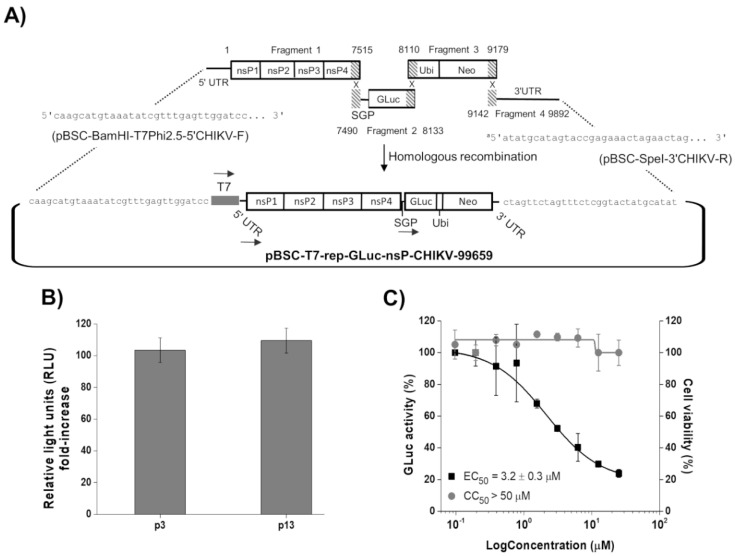
Development and characterization of the BHK-21-GLuc-nsP-CHIKV-99659 cell line. (**A**) Schematic representation of rep-GLuc-nsP-CHIKV-99659 construction. The ligation of fragments 1 and 4 to the pBSC-HDR vector was driven by homologous sequences inserted by the oligonucleotides pBSC-BamHI-T7Phi2.5-5′CHIKV-F and pBSC-SpeI-3′CHIKV-R, respectively (see Table 1). The dashed areas correspond to the overlapping regions between the fragments. The numbers in fragment 1 (1 to 7515 nt), 2 (7490 to 8133 nt), 3 (8110 to 9179 nt), and 4 (9142 to 9892 nt) correspond to the positions in rep-GLuc-nsP-CHIKV-99659. The arrows correspond to transcription driven by the T7 RNA polymerase promoter and the CHIKV genomic (at 5′UTR) and subgenomic promoters (SGP). ^a^ The sequence in pBSC-SpeI-3′CHIKV-R corresponds to the reverse complement (Table 1). (**B**) Comparison of GLuc activity between passages 3 and 13 of the BHK-21-GLuc-nsP-CHIKV-99659 cell line. The cells’ supernatants were subjected to luciferase activity assay to test replicon stability throughout cultivation. (**C**) Antiviral assays of suramin in a 96-well plate format. Replicon cells were incubated with the inhibitor in a serial dilution for 48 h and both the GLuc signal (black squares) and cell viability (gray circles) were measured from the supernatant. Average results of two independent experiments. Error bars represent standard deviations.

The CHIKV replicon expressing GLuc and Neo sequences was successfully developed by homologous recombination of four DNA fragments in yeast cells (Figure 1A). BHK-21-GLuc-nsP-CHIKV-99659 cell line was obtained after transfection with in vitro transcribed replicon RNA and antibiotic selection. To assess the replicon stability, we compared the GLuc activity of BHK-21-GLuc-nsP-CHIKV-99659 cells in different passages: one corresponding to the third post-selection culture (p3) and another to passage 13 (p13). The GLuc activity signals were very similar between passages, confirming the maintenance of the BHK-21-GLuc-nsP-CHIKV-99659 phenotype throughout the cultivation (Figure 1B).

Using suramin, we evaluated whether the CHIKV replicon cells would be suitable for the HTS in a 96-well format. As shown in Figure 1C, the compound inhibited GLuc activity in a concentration-dependent manner, with an EC_50_ value of 3.2 ± 0.3 µM, similar to that previously described using an in vitro assay with a replication-transcription complex (RTC) isolated from CHIKV replicon-transfected cells (EC_50_ of 6.7 µM) [31]. Moreover, no cytotoxicity was observed up to 50 µM of the inhibitor.

### 3.2. Identification of ITZ, GSK-983, Rubitecan, and MMV676270 as Inhibitors of CHIKV Replicon Replication

**Figure 2:**
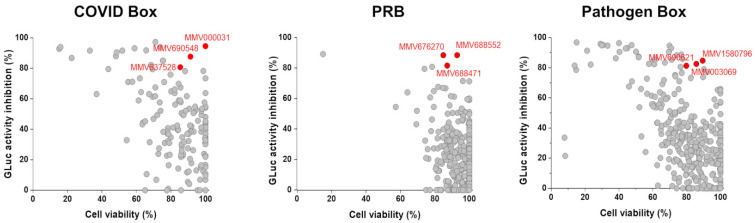
Replicon-based HTS for the COVID Box, Pandemic Response Box (PRB), and Pathogen Box. Scatter plots for the screening results of the 960 compounds in the primary screenings at 10 µM. In y-axis, the relative GLuc activity inhibition, and in x-axis, the relative cell viability. Selected compounds are highlighted in red and identified by their MMV code.

The replicon-based HTS were performed using 1% DMSO as a negative control and suramin as a positive control. Of the total 960 tested compounds, 55 inhibited the luciferase signals in ≥80%, being 17 from the COVID Box, 33 from the PRB, and 5 from the Pathogen Box (Figure 2). However, only 9 out of those 55 molecules, 3 from each library, exhibited cell viability ≥80% at 10 µM (Figure 2).

**Figure 3:**
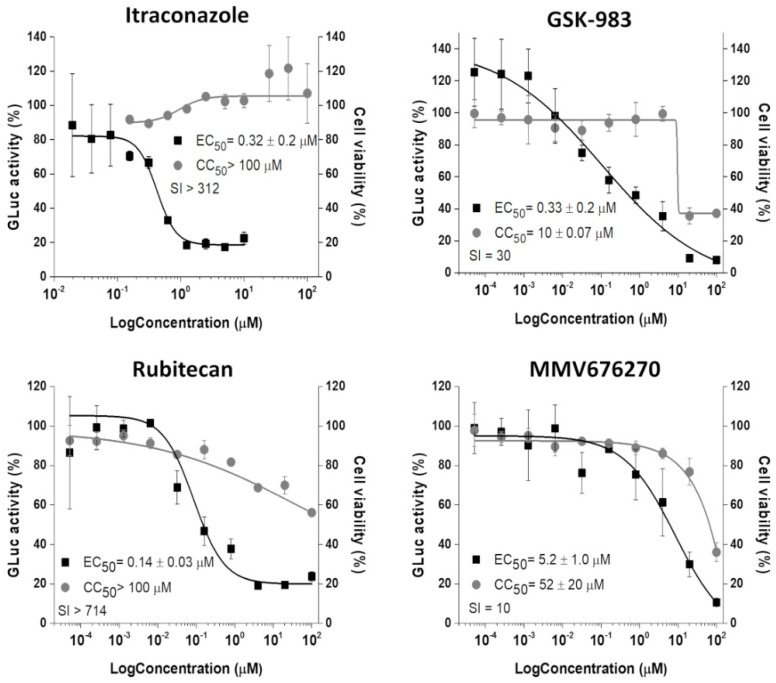
Concentration–response curves (EC_50_ and CC_50_) of selected compounds. The CHIKV replicon cells were treated with compounds at 2-fold (Itraconazole) or 5-fold (GSK-983, rubitecan, and MMV676270) serial dilutions for 48 h. GLuc signal (black squares) was measured from the supernatant, while cell viability (gray circles) was measured employing MTT assay. Average results of two independent experiments. Error bars represent standard deviations.

Those 9 selected molecules were evaluated in a concentration-dependent manner and 4 of them, itraconazole (ITZ-MMV637528), GSK-983 (MMV690621), rubitecan (MMV1580796), and MMV676270, showed antiviral activities with EC_50_ values at a nano to low micromolar range (Figure 3). GSK-983 and MMV676270 displayed moderate toxicities to the cells, while ITZ and rubitecan were not toxic up to 100 µM, resulting in SI values ranging from 10 to >714.

Based on the results obtained for ITZ, we additionally tested a panel of ten clinically used azoles in the primary screenings; however, none of them inhibited the GLuc signals in more than 80%, although they did not show significant toxicities (Table 2).

### 3.3. ITZ Strongly Inhibits CHIKV Infection In Vitro

**Figure 4:**
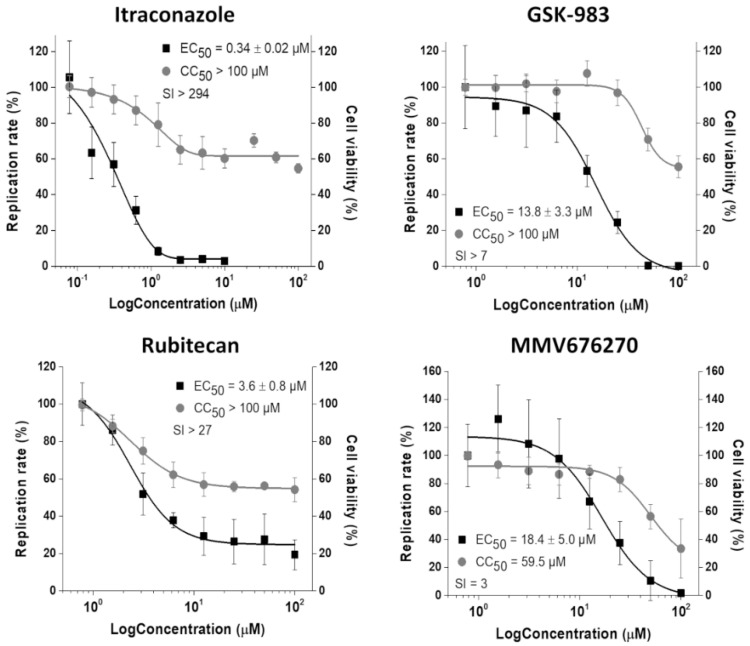
Inhibition of CHIKV-*nanoluc* infection in vitro. Concentration–response curves of selected compounds showing the relative replication rate (y-axis) over the compound concentration in log scale (x-axis). Average results of three independent experiments, each measured in triplicates, are shown, and error bars represent standard deviations.

Using CHIKV-*nanoluc*, which express *nanoluciferase* as a reporter, we further characterized the antiviral activity of the four inhibitors selected by the replicon-based assay. BHK-21 cells were infected with CHIKV-*nanoluc* at a MOI of 0.1 and simultaneously treated with two-fold serial dilutions of the compounds. As a result, the treatment with GSK-983, rubitecan, and MMV676270 decreased the viral replication with EC_50_ values at a low micromolar range (Figure 4). MMV676270 displayed a CC_50_ of 59.5 µM, resulting in SI of 3, while GSK-983 and rubitecan were not toxic to BHK-21 cells up to 100 µM, resulting in SI values of >7 and >27, respectively. Notably, ITZ was the most potent antiviral molecule, showing an EC_50_ of 0.34 ± 0.02 µM with no cytotoxic effect on the cell viability up to 100 µM, and SI of >294 (Figure 4).

## 4. Discussion

CHIKV remains a potential threat to public health with no specific antiviral available [9]. In this study, we successfully constructed a replicative CHIKV reporter replicon using homologous recombination in yeast, a strategy previously used to obtain reverse genetics systems for RNA viruses, such as dengue, yellow fever, bovine viral diarrhea virus and infectious bursal disease virus (IBDV) [29,32,33,34]. A BHK-21 cell line expressing the GLuc-Neo-CHIKV system, the BHK-21-GLuc-nsP-CHIKV-99659, was developed and demonstrated to persistently express the replicon RNA with no change in GLuc activity over 10 passages. Using replicon-based HTS we evaluated 960 MMV/DNDi compounds and identified rubitecan, GSK-983, MMV676270, and itraconazole with specific anti-CHIKV activities, which were confirmed in viral infection assays using the recombinant CHIKV-*nanoluc*.

The antiviral rubitecan, a camptothecin analog known to inhibit topoisomerase I [35], exhibited a very high SI against CHIKV on replicon-based assays, though this value was considerably lower on the viral infection assays. This compound is orally available, well tolerated, and was shown to display an anti-HIV effect in infected peripheral blood lymphocytes (PBLs) [36], evidencing its potential to be further studied as an inhibitor of other RNA viruses, such as CHIKV. Another antiviral identified was GSK-983, a tetrahydrocarbazole inhibitor of the dihydroorotate dehydrogenase (DHODH) [37]. DHODH and other enzymes of the pyrimidine biosynthesis pathway are investigated as targets of broad-spectrum antivirals, including the ones with anti-CHIKV activities such as atovaquone, RYL-634, and DD363, but targeting host factors may interfere with cell viability (reviewed in [38]). This could explain the lower CC_50_ observed in replicon-based assays, in which cells are incubated with the compound for a longer period of time than that for the viral infection assays. Compound MMV676270 exhibited the lowest SI among the 4 inhibitors and is not a well-studied molecule with only a reported activity against *Plasmodium falciparum* and *P. berghei* in the chemical database PubChem. The discrepancies between the EC_50_ values for the three compounds resulting from the two different cell-based assays are not surprising because of intrinsic differences among these assays. As an example, the RNA replication levels in replicon-containing cells may differ from that in virus-infected cells, contributing to the system-to-system variation of efficacy (EC_50_) obtained for a given compound [39,40]. Nevertheless, our results clearly demonstrate that rubitecan, GSK-983, and MMV676270 effectively inhibit CHIKV replication in vitro.

The most noteworthy compound identified was itraconazole (ITZ), a broad-spectrum antifungal agent. With an oral bioavailability of 55%, ITZ is a safe and cheap drug that allows long-period treatments of up to 12 months and regimen doses of up to 200 mg twice daily, making it an ideal candidate for repurposing, thus reducing the costs of developing new drugs against CHIKV [41,42]. Several studies have described in vitro activities of ITZ against +ssRNA viruses, such as enteroviruses, echovirus 30, dengue virus, and SARS-CoV-2 [43,44,45,46,47]. Although its precise mechanism of action has not been elucidated yet, in enteroviruses ITZ was shown to inhibit viral RNA replication by targeting oxysterol-binding protein (OSBP), which is responsible for trafficking of cholesterol and phosphatidylinositol-4-phosphate between membranes, therefore affecting the formation of the replication organelles [43,47]. The antiviral effect of ITZ could be, in part, based on such mechanism, but it is conceivable that it acts on viral replication via multiple mechanisms [45]. Our results also show that the anti-CHIKV effects are exclusive for this member of the azole series.

Recently, Posaconazole (PCZ), a structural analog of ITZ, was identified as a potent inhibitor of alphaviruses replication, showing comparable levels of Semliki Forest virus (SFV) replication inhibition when added at the time of inoculation or at 3 h post-inoculation (h.p.i), suggesting that this molecule acts on entry or early post-entry steps in the viral life cycle. Moreover, PCZ showed no toxic effects up to 100 µM concentration [48]. These findings are in agreement with the anti-CHIKV activity identified herein for ITZ but not for PCZ, as replicon-based screenings allow only the discovery of molecules affecting RNA replication, but not viral entry or assemble/release [12]. In conclusion, our results show that ITZ is a potent inhibitor of CHIKV replication and bring more attention to the potential use of antifungal triazoles as broad-spectrum antivirals. More studies are needed to confirm the *in vivo* efficacy of ITZ treatment on CHIKV infections.

## Figures and Tables

**Table 1:** Oligonucleotides used to construct rep-GLuc-nsP-CHIKV-99659.

Oligonucleotide	Sequence (5′-3′)	Amplicon
pBSC-BamHI-T7Phi2.5- 5′CHIKV-F ^a^	^b,c^**CAAGCATGTAAATATCGTTTGAGTTGGATCC**CAGTAATACGACTCACTATTATGGCTGCGTGAGACACACGTAG	Fragment 1 (T7 RNA polymerase promoter; CHIKV 5′UTR and nsP1-nsP4)
CHIKV-7515R	**GCAAAATAGGTAGCTGTAGTGCGTAC**CTATTTAGGACCGCCGTACAAG	
CHIKV1-GLuc-F	^d^***GTACGCACTACAGCTACCTATTTTGC****AAAAGCCGACAGCAGGTACCTAAATACCAATCAGCCATA*ATGGGAGTCAAAGTTCTGTTTGCCCTG	Fragment 2 (CHIKV subgenomic promoter and *Gaussia* luciferase gene)
GLuc-Ubiq-R	**CACGAAGATCTGCATGTTTAAACC**GTCACCACCGGCCCCCTTGATC	
Ubiq-F	**GGTTTAAACATGCAGATCTTCGTG**AAG	Fragment 3 (Ubiquitination sequence and neomycin phosphotransferase gene)
CHIKV1-Neo-R	**CTTTAGGGACGCGTATGCCTTCATACCTAGTTGTCAAG**TCAGAAGAACTCGTCAAGAAGGCGATAG	
CHIKV-3UTR-F	**CTTGACAACTAGGTATGAAGGCATAC**	Fragment 4 (CHIKV 3′UTR)
pBSC-SpeI-3′CHIKV-R	**ATATGCATAGTACCGAGAAACTAGAACTAG**TTTTTTTTTTTTTTTTTTTTTTTTTTTTTTTTTTTTTTTTGAAATATTAAAAACAAAATAACATCTCC	

^a^ Oligonucleotides pBSC-BamHI-T7Phi2.5-5′CHIKV-F and CHIKV-3′UTR-R (5′-TTTTTTTTTTTTTTTTTTTTTTTTTTTTTTTTTTTTTTTTTGAAATATTAAAAACAAAATAACATCTCCTACGTCCCTATGGGTAC-3′) were used in full-length PCR; ^b^ Nucleotides used for homologous recombination are in bold; ^c^ The T7 RNA polymerase promoter is underlined; ^d^ The CHIKV subgenomic promoter is in italics.

**Table 2:** Activity of azoles against CHIKV replicon replication.

Compound	GLuc Inhibition	Cell Viability
Voriconazole	13.9%	91.8%
Econazole	43.8%	81.8%
Tioconazole	54.8%	68.4%
Clotrimazole	70.7%	70.2%
Ketoconazole	0%	100%
Fluconazole	0%	94.3%
Posaconazole	11.8%	100%
Ravuconazole	25.4%	100%
Isavuconazole	28.6%	100%
Miconazole	27.8%	100%

The luciferase activity inhibition and cell viability of the ten azoles evaluated in the primary replicon-based screenings at 10 µM are shown.

## Data Availability

The data presented in this study are openly available in PubMed Central at [10.3390/v14071351].
